# Targeted therapy in thyroid cancer: molecular alterations and clinical management

**DOI:** 10.3389/fendo.2026.1810813

**Published:** 2026-06-05

**Authors:** YiHeng Yang, YeSheng Zhang, YongCan Xu, XiaoXin Gu, Neng Lou, GuoChao Ye

**Affiliations:** 1 Department of General Surgery, Huzhou Central Hospital, Fifth School of Clinical Medicine of Zhejiang Chinese Medical University, Huzhou, Zhejiang, China; 2 Department of General Surgery, Affiliated Huzhou Hospital, Zhejiang University School of Medicine, Huzhou, Zhejiang, China; 3 Department of General Surgery, Huzhou Central Hospital, Affiliated Central Hospital of Huzhou University, Huzhou, Zhejiang, China

**Keywords:** molecular alterations, precision medicine, radioiodine-refractory thyroid cancer, targeted therapy, thyroid cancer

## Abstract

**Background:**

This review summarizes key actionable molecular alterations in thyroid cancer and examines how molecular profiling can be translated into practical, subtype-specific targeted treatment strategies for advanced, recurrent, or radioiodine-refractory disease.

**Methods:**

A narrative synthesis was performed using evidence from landmark clinical trials, current consensus guidelines, and representative translational studies on thyroid cancer drivers and targeted therapies. Molecular targets were categorized by signaling pathways and pathological subtypes (DTC, PTC/FTC, ATC/PDTC, and MTC), with emphasis on clinical indications, efficacy signals, and toxicity profiles.

**Results:**

The identification of specific genomic drivers, such as BRAF V600E, RET mutations/fusions, and NTRK fusions, has revolutionized the management of thyroid cancer. Precision highlights include the use of BRAF/MEK inhibitors in BRAF-mutant ATC and highly selective RET or TRK inhibitors for fusion-positive tumors. For cases lacking these specific markers, VEGFR-targeted multikinase inhibitors (e.g., lenvatinib) remains the standard of care for RAIR-DTC. Furthermore, “redifferentiation” therapies show promise in restoring radioiodine sensitivity, while emerging pathways like PI3K/AKT/mTOR and immune checkpoints offer new avenues for combination therapy to overcome treatment resistance.

**Conclusions:**

The therapeutic paradigm in thyroid cancer is shifting from non-selective multikinase inhibition toward molecularly matched, combination-based, and adaptively sequenced strategies. Early and comprehensive genomic profiling—including fusion detection—is essential to optimize treatment selection, address resistance, and expand precision therapy options across disease subtypes.

## 1 Background

Thyroid cancer is the most common malignant tumor of the endocrine system. With the widespread large-scale application of imaging examinations and fine-needle aspiration biopsy, its incidence has increased rapidly over the past decade. Among all patients, epidemiological data indicate that the ratio of female to male patients can be as high as 3:1 (1). Thyroid cancer is generally classified into differentiated thyroid cancer (DTC), medullary thyroid carcinoma (MTC), and anaplastic thyroid carcinoma (ATC). DTC is further subdivided into papillary thyroid carcinoma (PTC), follicular thyroid carcinoma (FTC), and oncocytic carcinoma. Among these subtypes, DTC originates from thyroid follicular cells, whereas MTC arises from parafollicular C cells of the thyroid gland (2).

The majority of differentiated thyroid cancers exhibit favorable biology and prognosis; however, in a subset of patients, prognosis is significantly worsened due to tumor aggressiveness, insensitivity or resistance to radioactive iodine (RAI) therapy, or the presence of distant metastases (3). Current standard treatment strategies mainly include surgical resection, radioactive iodine therapy, external beam radiotherapy, and multikinase tyrosine kinase inhibitors (TKIs) (4). However, these conventional approaches still face limitations in the management of some high-risk, advanced, or recurrent metastatic cases, including limited therapeutic efficacy, substantial adverse effects, and marked interindividual variability, making it difficult to meet the requirements of precision therapy (3, 5).

With advances in tumor molecular biology research, driver gene mutations and associated signaling pathways in thyroid cancer have been progressively elucidated, including alterations involving BRAF, RET, NTRK, RAS, and TERT promoter mutations. These findings have provided a solid foundation for the development of targeted therapies. By directly intervening in key processes such as tumor growth, differentiation, angiogenesis, and immune evasion, targeted therapy has become an important adjunct in the treatment of advanced thyroid cancer, demonstrating notable efficacy particularly in RAIR-DTC, ATC, and MTC (6).

In view of the rapid development and broad clinical application prospects of targeted therapy in thyroid cancer, this review aims to comprehensively summarize currently known molecular targets and their corresponding targeted therapeutic strategies. Both common and emerging molecular targets are covered, with a brief overview of their biological characteristics, representative targeted agents, indications, and clinical efficacy, in order to provide a comprehensive reference for clinical decision-making and future research.

### 1.1 Literature search and selection strategy

This article was conducted as a narrative review. Relevant literature was identified through searches of PubMed, Web of Science, and Google Scholar, using combinations of terms including “thyroid cancer”, “targeted therapy”, “molecular alterations”, “radioiodine-refractory differentiated thyroid cancer”, “anaplastic thyroid cancer”, “medullary thyroid cancer”, “BRAF”, “RET”, “NTRK”, “VEGFR”, “PI3K/AKT/mTOR”, “redifferentiation”, and “immune checkpoint”. Priority was given to landmark clinical trials, current guidelines and consensus statements, high-quality reviews, and representative translational or mechanistic studies directly related to molecular targets and targeted therapeutic strategies in thyroid cancer. Studies were selected based on their relevance to actionable alterations, therapeutic efficacy, toxicity profiles, resistance mechanisms, molecular testing, and clinical decision-making. Articles not directly related to thyroid cancer targeted therapy, duplicate reports, or studies with limited relevance to the scope of this review were excluded. Because this article is a narrative review, no formal meta-analysis, systematic risk-of-bias assessment, or quantitative evidence synthesis was performed.

## 2 Classification of molecular targets and therapeutic strategies

In recent years, research on targeted therapy for thyroid cancer has focused on multiple key oncogenic pathways and driver alterations, including the MAPK pathway, RET/NTRK fusions, the PI3K/AKT/mTOR pathway, angiogenic pathways, and immune checkpoints. The distribution of molecular alterations varies among different pathological subtypes. PTC is predominantly characterized by the BRAF V600E mutation (1), MTC is most characteristically associated with RET mutations (7, 8), whereas ATC commonly exhibits multiple concurrent genetic alterations accompanied by activation of immune evasion mechanisms (6).

**Figure 1:**
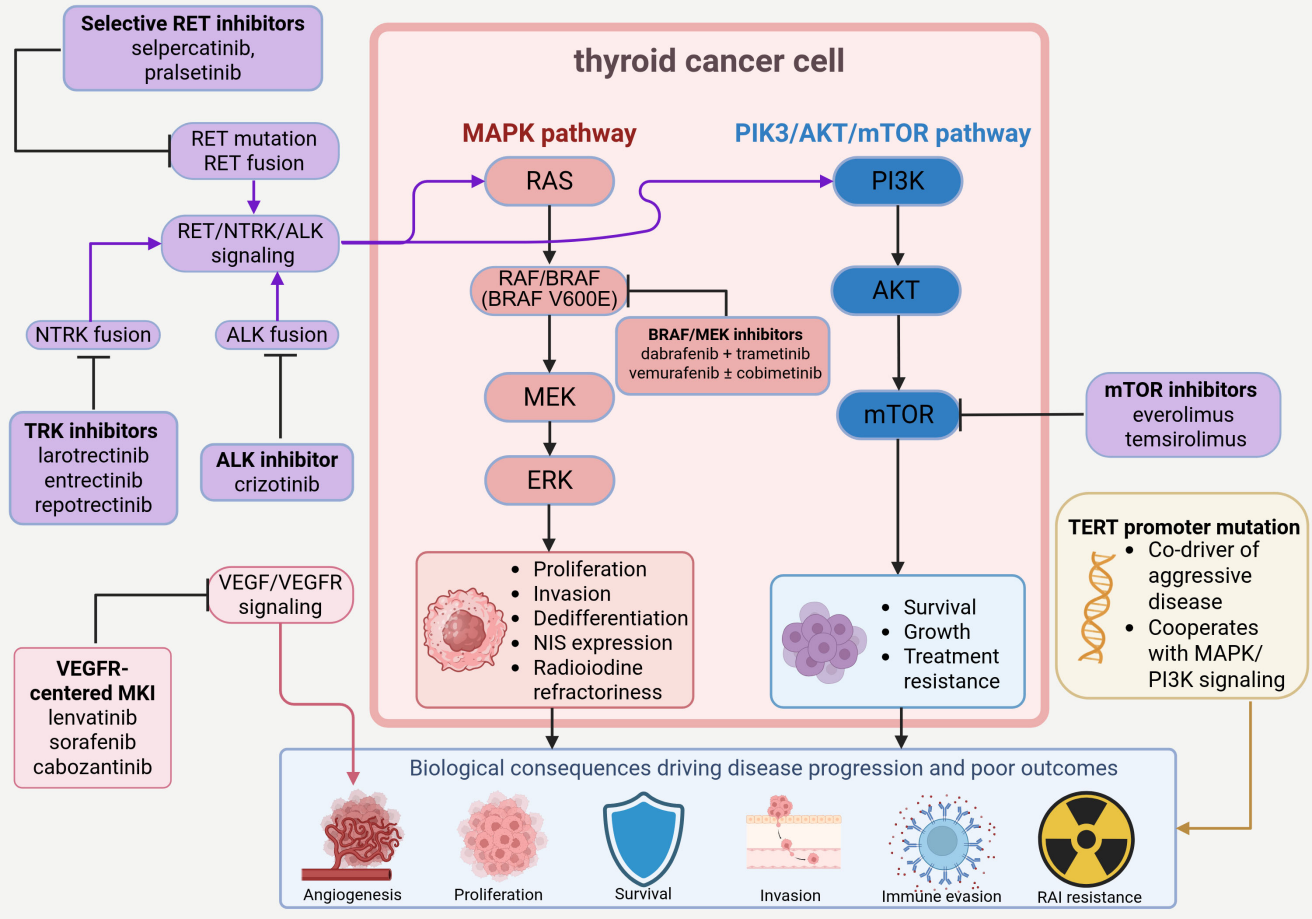
Molecular targets and signaling pathways involved in targeted therapy for thyroid cancer. This schematic summarizes the major molecular alterations, signaling pathways, representative targeted agents, and biological consequences involved in thyroid cancer progression and targeted therapy.

**Figure 2:**
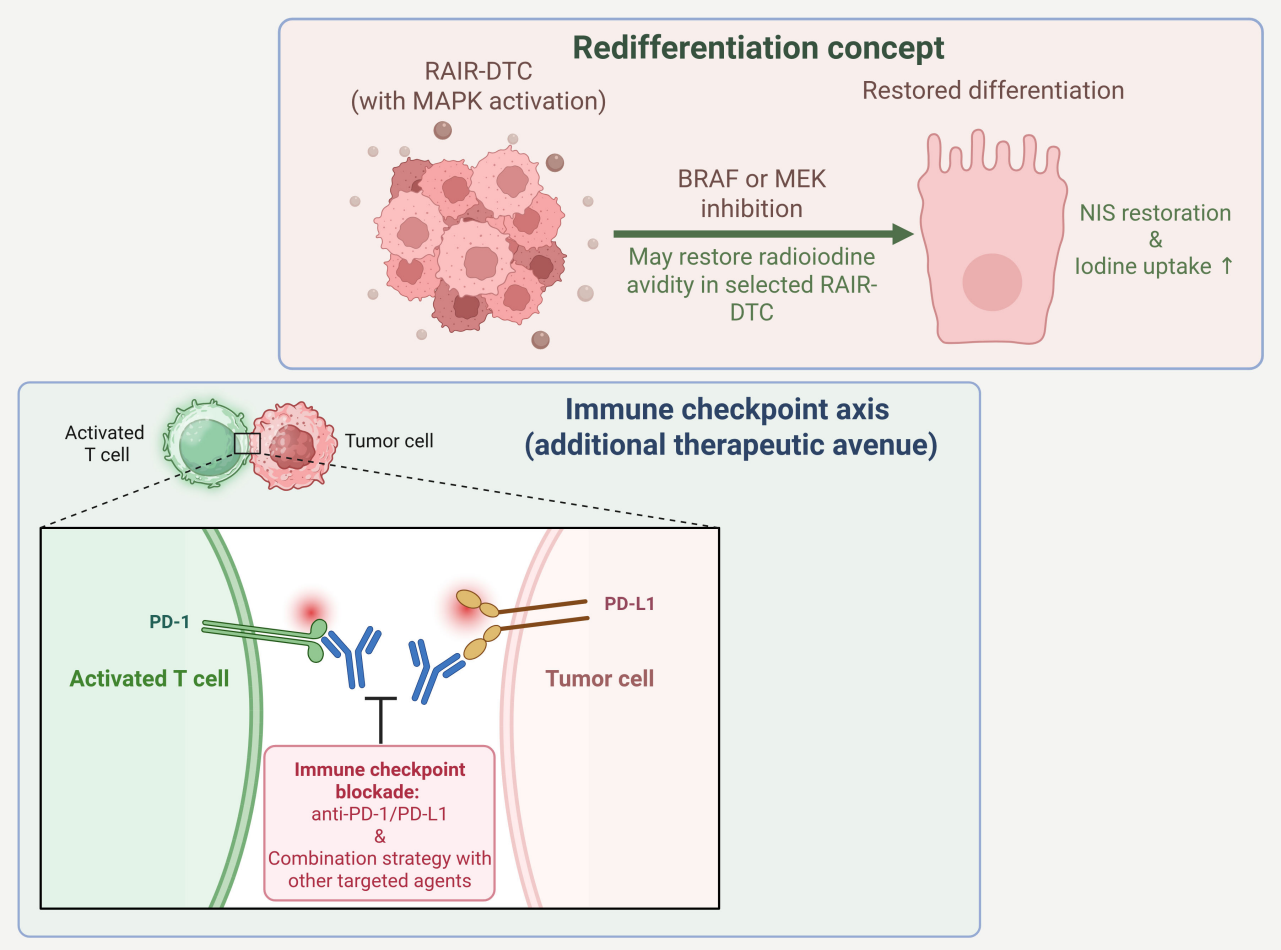
Redifferentiation therapy and immune checkpoint blockade as additional therapeutic strategies in thyroid cancer. This schematic illustrates the concepts of MAPK pathway inhibition–mediated redifferentiation and PD-1/PD-L1 immune checkpoint blockade, highlighting their potential roles as complementary therapeutic strategies in advanced thyroid cancer.

**Table 1** summarizes representative molecular targets in thyroid cancer, their oncogenic mechanisms, representative agents, and the corresponding indicated cancer subtypes. **Figures 1** and **2** provide an integrated schematic overview of the major molecular targets, signaling pathways, biological consequences, and corresponding therapeutic strategies in thyroid cancer.

**Table 1:** Key molecular targets and pathways in thyroid cancer: mechanisms, representative agents, and clinical indications.

Target/pathway	Oncogenic mechanism	Representative agents	Applicable thyroid cancer subtypes
BRAF V600E	Constitutive activation of the MAPK pathway, promoting proliferation and anti-apoptosis; associated with dedifferentiation and radioiodine (RAI) resistance.	Dabrafenib Vemurafenib	PTC, ATC
RET mutations/fusions	Activation of downstream RAS/MAPK and PI3K signaling pathways, driving cellular transformation.	Selpercatinib Pralsetinib	MTC, PTC (with RET fusions)
NTRK fusions	Activation of multiple oncogenic signaling pathways (MAPK/PI3K), promoting tumorigenesis.	Larotrectinib Entrectinib	PTC and rare fusion-positive subtypes
RAS mutations	Activation of MAPK and PI3K pathways, enhancing cellular proliferation; more frequent in follicular carcinoma.	No specific targeted agents. Influences TKI selection	FTC, a subset of RAIR-DTC
VEGFR pathway	Promotes angiogenesis, tumor nutrient supply, invasion, and metastasis.	Lenvatinib Sorafenib Cabozantinib; Sunitinib	RAIR-DTC, ATC, MTC; selected advanced thyroid cancers
ALK fusions	Activation of ALK-related signaling pathways; identified in a small subset of PTC.	Crizotinib (investigational)	Rare PTC cases
PI3K/AKT/mTOR pathway	Promotes cell growth, metabolism, and anti-apoptotic signaling; frequently dysregulated in ATC.	Everolimus Temsirolimus (investigational)	ATC, PDTC (selected studies)
EGFR family	Overexpression enhances tumor growth and metastasis; more common in ATC.	Afatinib (investigational)	ATC
TERT promoter mutations	Upregulation of telomerase activity, promoting cellular immortalization; often co-occurs with BRAF mutations.	No direct targeted agents; prognostic biomarker	PTC, ATC, FTC
PD-1/PD-L1 axis	Suppression of T-cell function, facilitating immune evasion; frequently observed in undifferentiated carcinoma.	Nivolumab Pembrolizumab (immunotherapy)	ATC, PDTC (selected studies)

## 3 Description of molecular targets

### 3.1 The MAPK pathway (RAF–MEK–ERK axis and related fusions and mutations)

The MAPK pathway is one of the most central and most common oncogenic signaling axes in thyroid cancer and plays a dominant role particularly in PTC. Sustained activation of this pathway can be achieved through BRAF mutations, RAS mutations, or fusions involving receptor tyrosine kinases such as RET, NTRK, and ALK, thereby driving cell proliferation, invasion, metastasis, and dedifferentiation; it is also closely associated with RAI resistance, “redifferentiation therapy,” and various targeted combination strategies (9, 10).

#### 3.1.1 BRAF V600E

The BRAF gene is located on chromosome 7 and encodes the B-Raf protein, a key component of the mitogen-activated protein kinase (MAPK) signaling pathway (11). BRAF V600E is the most common mutation of this gene and refers to the substitution of valine (Val) by glutamic acid (Glu) at codon 600, resulting in constitutive activation of the BRAF protein. This leads to persistent activation of the MAPK/ERK pathway independent of upstream signaling, thereby promoting tumor cell proliferation, metastasis, and resistance to apoptosis (11, 12). This mutation is one of the most important driver gene alterations in thyroid cancer and is particularly prevalent in PTC, with a reported positivity rate of approximately 40%–60% (11, 13). Moreover, studies have shown that BRAF V600E is highly prevalent in the Han Chinese population (14).

Although BRAF V600E has been frequently associated with aggressive clinicopathological features, including lymph node metastasis and tumor recurrence (13, 15), its prognostic impact is not uniform across all clinical contexts and should not be interpreted as an isolated universal marker of poor outcome. Increasing evidence suggests that the most consistent and clinically meaningful association with adverse prognosis is observed when BRAF V600E coexists with TERT promoter mutations. The coexistence of BRAF V600E and TERT promoter mutations has been shown to identify a subgroup of PTC with particularly aggressive behavior, higher recurrence risk, and increased disease-specific mortality (16, 17). Therefore, BRAF V600E status should be evaluated together with TERT promoter mutation status and other molecular or clinicopathological factors for more accurate risk stratification.

In addition, BRAF V600E is closely associated with radioactive iodine (RAI) refractoriness, potentially through suppression of the expression and membrane localization of the sodium/iodide symporter (NIS), thereby impairing effective radioactive iodine uptake by tumor cells and leading to loss of sensitivity to RAI therapy (18–20). These observations provide a biological rationale for BRAF-targeted therapy in selected patients with RAI-refractory PTC, although its clinical role should be interpreted according to disease subtype, treatment setting, and available evidence (12, 21).

Currently, representative agents targeting this alteration include dabrafenib and vemurafenib, both of which are BRAF kinase inhibitors (12). Both agents have been applied in the treatment of various BRAF V600E–mutated malignancies, including melanoma and non–small cell lung cancer (NSCLC) (12, 22). In thyroid cancer, particularly in RAI-refractory PTC and ATC, BRAF inhibitors have shown antitumor activity when used as monotherapy or in combination regimens, although the strength of evidence and regulatory status differ substantially between disease settings (22, 23). For example, dabrafenib in combination with the MEK inhibitor trametinib has received accelerated approval from the U.S. Food and Drug Administration (FDA) for the treatment of BRAF V600E–mutant ATC, representing an established approved regimen in this specific setting. More recently, combination strategies integrating BRAF-targeted therapy with immunotherapy have attracted increasing attention in ATC. In a nonrandomized phase II clinical trial, Cabanillas, M.E. et al. evaluated mutation-directed targeted therapy combined with the anti–PD-L1 antibody atezolizumab in ATC. In the BRAF V600E cohort, patients received vemurafenib plus cobimetinib combined with atezolizumab, achieving an objective response rate of 50%, a median progression-free survival of 13.93 months, and a median overall survival of 43.24 months, suggesting that BRAF/MEK inhibition plus immune checkpoint blockade may provide a potential combination strategy for selected patients with BRAF-mutant ATC, although this approach still requires further prospective validation (24). In addition, the FAST multidisciplinary group consensus statement by Hamidi S et al. emphasized the importance of rapid BRAF V600E testing and timely initiation of BRAF-directed therapy in BRAF V600E-variant ATC. The consensus favored dabrafenib plus trametinib combined with pembrolizumab rather than dabrafenib plus trametinib alone for stage IVB/IVC BRAF V600E-variant ATC when feasible, while also considering the addition of pembrolizumab at progression as an acceptable strategy; however, this recommendation should be distinguished from randomized phase III evidence and interpreted in the context of expert consensus and emerging clinical data (25).

In selected patients with RAI-refractory PTC, BRAF inhibitors may induce re-expression of NIS and restore radioactive iodine uptake, thereby enabling “redifferentiation” therapy (19, 26), which offers some patients the possibility of re-treatment with RAI.

#### 3.1.2 HRAS, NRAS, and KRAS mutations

The RAS family includes three members—HRAS, NRAS, and KRAS—all of which encode small GTPases located upstream of the MAPK and PI3K signaling pathways and play a central regulatory role in controlling cellular proliferation, differentiation, and apoptosis (27, 28). RAS proteins are activated in the GTP-bound state and can stimulate sustained activation of downstream pathways such as RAF–MEK–ERK and PI3K–AKT–mTOR (27). When point mutations occur in RAS genes (e. g., NRAS Q61R, KRAS G12D), RAS proteins are unable to efficiently hydrolyze GTP and remain constitutively active, thereby driving tumor initiation and progression (28). In contrast to the strong and sustained MAPK activation induced by BRAF V600E, RAS mutations in thyroid cancer often exhibit a parallel activation pattern involving both MAPK and PI3K pathways, suggesting a potential association with bypass signaling activation and the development of therapeutic resistance (9).

In thyroid cancer, RAS mutations are mainly observed in FTC and follicular variant papillary thyroid carcinoma (FV-PTC), and can also be detected in ATC as well as in a subset of RAI-refractory differentiated thyroid cancers (29, 30). Among these, NRAS is the most common mutational subtype (31), with a mutation frequency of approximately 40% in FTC (29). RAS mutations are generally associated with lower MAPK pathway activity, and the tumor biological behavior driven by RAS alterations differs from that of BRAF-mutant tumors, showing a greater tendency toward hematogenous metastasis rather than lymph node metastasis (29).

Overall, tumors harboring RAS mutations may retain relatively better sensitivity to radioactive iodine therapy, although they may still progress to RAI-refractory disease (26, 30). In addition, RAS mutations frequently coexist with TERT promoter mutations, and such co-mutations are indicative of a significantly unfavorable prognosis (32).

At present, no highly selective targeted agents directed specifically against RAS mutations have been approved for use in thyroid cancer. Multikinase TKIs such as lenvatinib and sorafenib remain standard systemic options for progressive RAIR-DTC regardless of RAS status; however, the association between therapeutic efficacy and RAS mutational status remains unclear (26, 30, 33). In recent years, targeted agents against specific KRAS mutations (e. g., G12C), such as sotorasib, have shown progress in other malignancies (34, 35), but their application in thyroid cancer remains at the investigational stage. Other studies have focused on indirectly targeting downstream pathways of RAS signaling, such as MEK inhibitors, although the overall therapeutic benefit has been limited (27, 36), and RAS mutations currently serve mainly as molecular classifiers and prognostic or redifferentiation-related biomarkers rather than direct therapeutic indications; treatment recommendations are largely extrapolated from broader RAIR-DTC cohorts rather than RAS-specific prospective trials.

#### 3.1.3 RET mutations and fusions

The RET (rearranged during transfection) gene is located on chromosome 10 and encodes a transmembrane receptor tyrosine kinase (8, 37). This receptor plays an important role in the proliferation and differentiation of neural crest cells during embryonic development (8). The oncogenic mechanisms of RET mainly include two forms—point mutations and gene fusions—both of which lead to aberrant enhancement of its tyrosine kinase activity, thereby activating downstream signaling pathways such as MAPK and PI3K/AKT and promoting tumor cell growth, migration, and survival (8, 38).

In thyroid cancer, RET mutations are most commonly observed in MTC (7). Approximately 60% of sporadic MTC cases harbor activating RET mutations, whereas in familial MTC or multiple endocrine neoplasia type 2 (MEN2), the prevalence is even higher, exceeding 95% (39, 40). RET point mutations, particularly RET M918T, are closely associated with high tumor aggressiveness and poor prognosis (41, 42).

In contrast, RET fusions (e. g., RET/PTC) mainly occur in PTC and are especially common in pediatric patients and individuals with prior radiation exposure (43, 44). RET fusion genes result from the juxtaposition of the RET tyrosine kinase domain with promoter or regulatory regions of other genes, leading to constitutive activation and malignant cellular transformation (8, 44).

Targeted therapies against RET activation have achieved significant advances in recent years, although the strength of evidence differs between RET-mutant MTC and RET fusion–positive DTC. Selpercatinib (LOXO-292) and pralsetinib (BLU-667) are highly selective RET inhibitors that have been approved by the FDA for selected patients with RET-mutant MTC and RET fusion–positive RAI-refractory DTC (45, 46). Multiple clinical studies have demonstrated that selective RET inhibitors can achieve high response rates in RET-positive thyroid cancer, with generally more manageable toxicity profiles compared with conventional multikinase TKIs such as vandetanib and cabozantinib (39, 45). Importantly, the phase III LIBRETTO-531 trial directly compared selpercatinib as first-line therapy with physician’s choice of cabozantinib or vandetanib in patients with progressive, locally advanced or metastatic RET-mutant MTC who had not previously received kinase inhibitors. In this trial, selpercatinib significantly improved progression-free survival compared with the control group; the median progression-free survival was not reached in the selpercatinib group and was 16.8 months in the control group. Selpercatinib also improved treatment failure–free survival and achieved a higher overall response rate than cabozantinib or vandetanib, further supporting its role as a preferred first-line selective RET inhibitor in this specific population of advanced RET-mutant MTC (45).

In addition, RET inhibitors have also been applied in the treatment of other malignancies harboring RET fusions, such as non–small cell lung cancer, demonstrating cross-tumor activity that supports genotype-matched therapy across selected RET-altered tumors (46, 47). In the context of thyroid cancer, their use represents the clinical implementation of a “driver mutation–matched” precision treatment strategy. Nevertheless, durability of response may be limited by acquired resistance, including secondary RET kinase-domain mutations such as solvent-front alterations, and molecular re-profiling at progression is therefore important for subsequent treatment selection.

#### 3.1.4 NTRK fusions

The NTRK (neurotrophic tyrosine receptor kinase) gene family includes NTRK1, NTRK2, and NTRK3, which encode the neurotrophin receptors TrkA, TrkB, and TrkC, respectively. Under normal physiological conditions, these receptors are involved in the development, survival, and maintenance of nervous system function (48). NTRK fusions arise from chromosomal rearrangements that fuse the kinase domain of NTRK to promoter regions of other highly expressed genes, leading to constitutive expression and activation of Trk proteins. This results in activation of downstream signaling pathways such as MAPK, PI3K/AKT, and PLCγ, thereby driving tumorigenesis (49, 50).

In thyroid cancer, NTRK fusions are more frequently observed in PTC, particularly in pediatric patients, individuals with prior radiation exposure, or those with RAI-refractory disease (51, 52). Studies have shown that the prevalence of NTRK fusions in pediatric PTC can exceed 10%, whereas the frequency in adult PTC is relatively lower, at approximately 1%–2% (52, 53). NTRK fusions are considered driver alterations (50), and patients harboring these fusions may show substantial responses to highly selective NTRK inhibitors.

Currently, two highly selective NTRK inhibitors have been approved by the FDA for the treatment of solid tumors harboring NTRK fusions: larotrectinib and entrectinib (54). The initial approvals of these agents were tumor-agnostic, based on a pan-cancer indication for patients with unresectable or metastatic tumors lacking satisfactory alternative treatment options (54). Larotrectinib has demonstrated substantial antitumor activity in multiple phase I/II clinical trials, inducing rapid and durable responses in patients with NTRK fusion–positive RAIR-DTC and PTC, with objective response rates (ORR) exceeding 70%, including efficacy in RAIR-DTC, and with a generally manageable toxicity profile (55–57). However, thyroid cancer-specific evidence is mainly derived from relatively small cohorts within broader basket trials or case series, which limits direct randomized comparisons and precise estimates of long-term durability. Entrectinib is also a highly selective TRK inhibitor with additional activity against ROS1 and ALK, and is indicated for NTRK1/2/3 fusion–positive solid tumors. In pooled analyses. It has achieves an ORR of approximately 57% and demonstrates good control of central nervous system metastases, making it a potential option for patients at risk of brain involvement (58, 59). Given their manageable adverse event profiles, both agents have become recommended therapeutic options for NTRK fusion–positive thyroid cancer, supported by tumor-agnostic approvals, basket-trial evidence, and case-level thyroid cancer reports.

In addition, in 2024, the FDA approved the second-generation TRK inhibitor repotrectinib for the treatment of adult and pediatric patients aged 12 years or older with locally advanced or metastatic solid tumors harboring NTRK gene fusions. This agent was specifically developed to overcome resistance mediated by solvent-front mutations that emerge with first-generation inhibitors (60, 61). Repotrectinib is currently being evaluated across multiple solid tumors, including thyroid cancer, and has demonstrated potential for addressing acquired resistance, although thyroid cancer-specific clinical evidence remains limited (62).

It is noteworthy that although the overall incidence of NTRK fusions is low, they represent a rare but clinically valuable target for precision therapy. Consequently, genetic testing for NTRK fusions has increasingly been recommended in patients with RAIR-DTC or pediatric PTC, particularly in the absence of other known driver alterations.

#### 3.1.5 ALK fusions

ALK (anaplastic lymphoma kinase) is a receptor tyrosine kinase that was first identified in anaplastic large cell lymphoma (63, 64). Under physiological conditions, ALK expression is largely restricted to the nervous system during embryonic development and is minimal in adult tissues (65). When the ALK gene undergoes chromosomal rearrangements with genes harboring strong promoter activity (such as STRN or EML4), fusion genes (e. g., STRN–ALK, EML4–ALK) are generated. These fusions produce constitutively activated fusion proteins that drive persistent activation of downstream signaling pathways, including MAPK, PI3K–AKT, and JAK–STAT, thereby inducing malignant cellular transformation (63, 65, 66).

In thyroid cancer, the incidence of ALK fusions is relatively low; however, their clinical relevance has gained increasing attention (67, 68). ALK fusions are occasionally detected in PTC, but are reported more frequently in pediatric patients (69), individuals with RAI-refractory disease (70), and those with prior radiation exposure (71, 72). In addition, rare cases have been identified in ATC (73) and FTC (74). Given that ALK fusions represent “low-frequency but potentially actionable” driver events, early fusion testing may be considered in selected RAIR-DTC or ATC patients who are negative for conventional driver alterations or who exhibit rapid disease progression, in order to avoid missing potential opportunities for targeted therapy.

Targeted treatment against ALK fusions has been well established in NSCLC, where first-generation crizotinib, followed by alectinib, brigatinib, and lorlatinib, have been widely applied and have significantly improved patient survival (75, 76), however, these data should not be directly extrapolated to thyroid cancer without thyroid cancer-specific validation.

Although no ALK inhibitor has yet been specifically approved for thyroid cancer, case reports and preclinical studies suggest that crizotinib may produce antitumor activity or occasional tumor responses in thyroid cancer patients harboring STRN–ALK fusions (77, 78), suggesting an investigational or off-label precision treatment option for a small subset of patients. From a diagnostic perspective, ALK fusions represent structural variants; therefore, clinical detection relies on techniques capable of identifying gene fusions. Next-generation sequencing (NGS) panels that include fusion detection—particularly RNA-based fusion assays—are preferable when feasible, as they enable simultaneous identification of RET, NTRK, and ALK fusions as well as assessment of co-occurring mutations (68, 70); however, because thyroid cancer-specific therapeutic evidence remains limited and prospective trials are lacking, ALK-directed therapy should preferably be considered in the context of clinical trials or after multidisciplinary molecular tumor board assessment.

### 3.2 Angiogenic signaling pathway (the VEGF–VEGFR axis)

Angiogenesis represents a critical biological basis for sustained tumor growth and metastatic spread in advanced thyroid cancer. Activation of the VEGF/VEGFR axis promotes neovascularization and remodeling of the tumor microenvironment and has therefore become one of the most established and broadly applicable therapeutic pathways in systemic treatment of advanced thyroid cancer, including RAIR-DTC. Accordingly, multikinase TKIs commonly share inhibition of VEGFR as a central therapeutic mechanism (79, 80).

#### 3.2.1 VEGFR

Vascular endothelial growth factor (VEGF) and its receptors (VEGFRs) play pivotal roles in tumor angiogenesis and are key components of the tumor microenvironment that support sustained tumor growth, invasion, and metastasis (81, 82). In thyroid cancer—particularly in advanced DTC, ATC, and MTC—high expression of VEGF and VEGFR is frequently detected and is closely associated with increased tumor aggressiveness, radioactive iodine resistance, and poor prognosis (81, 83).

On this basis, multiple multikinase TKIs have been developed to target VEGFR and other tumor-related signaling molecules. These agents not only inhibit VEGFR-mediated angiogenesis but often concurrently target multiple kinases, including FGFR, PDGFR, RET, KIT, and BRAF, thereby exhibiting broad but less selective antitumor activity (82, 84). Representative agents include lenvatinib and sorafenib, both of which have been approved by the FDA and the National Medical Products Administration (NMPA) of China for the treatment of RAIR-DTC (85). Cabozantinib has also emerged as an important subsequent-line VEGFR-centered multikinase TKI for patients with RAIR-DTC who have progressed after prior VEGFR-targeted therapy (5, 86).

In the phase III randomized, placebo-controlled SELECT trial, lenvatinib significantly prolonged PFS in patients with RAIR-DTC to 18. 3 months, compared with 3. 6 months in the placebo group, with an ORR of 64. 8% (87–89). Sorafenib likewise demonstrated a clear PFS benefit in the DECISION trial, achieving a PFS of 10. 8 months versus 5. 8 months in the control group (85, 88). More recently, the phase III COSMIC-311 trial evaluated cabozantinib in patients with radioiodine-refractory DTC who had previously received lenvatinib and/or sorafenib. In this study, cabozantinib significantly improved progression-free survival compared with placebo, with a median PFS of 11.0 months versus 1.9 months, supporting cabozantinib as an evidence-based subsequent-line option after progression on earlier VEGFR-targeted therapy (86). However, these pivotal MKI trials were largely placebo-controlled rather than direct head-to-head comparisons between active agents; therefore, numerical PFS values should not be interpreted as direct evidence of superiority among lenvatinib, sorafenib, and cabozantinib. In addition, PFS improvement has not consistently translated into a clearly demonstrated overall survival advantage, partly because of crossover, subsequent therapies, and the relatively long natural history of some DTC cases.

In addition to these approved or more established agents, sunitinib has also been explored in DTC. Sunitinib is an oral multikinase inhibitor targeting VEGFRs, PDGFRs, KIT, and RET. Phase II studies in iodine-refractory or advanced DTC reported objective responses in a subset of patients and disease stabilization in a substantial proportion of cases, but these studies were generally small and non-comparative (90, 91). However, because the evidence for sunitinib in DTC remains mainly derived from phase II studies and lacks phase III confirmation, it is generally considered an investigational or alternative option rather than a standard first-line treatment for RAIR-DTC (90, 91).

The main adverse events associated with VEGFR-targeted TKIs include hypertension, proteinuria, decreased appetite, fatigue, and hand–foot skin reaction, which frequently necessitate close monitoring, dose interruption, dose reduction, or supportive management (92). In addition, lenvatinib has been explored in the treatment of ATC, particularly in patients without alternative therapeutic options, showing modest or context-dependent efficacy (93, 94), and studies combining lenvatinib with immunotherapy are currently underway (84, 95). For patients with MTC, multikinase TKIs such as cabozantinib and vandetanib are considered established options, particularly when selective RET inhibitors are unavailable, unsuitable, or after resistance develops (81, 96).

The advantage of multikinase TKIs lies in their ability to cover multiple oncogenic pathways, making them suitable for patient populations without identifiable highly actionable mutations or fusions. However, their broad target profiles are also associated with increased toxicity, underscoring the need for careful patient selection and proactive management of adverse effects. Selection among VEGFR-centered MKIs should integrate regulatory approval, line of therapy, pace of disease progression, tumor burden, comorbidities, expected toxicity, and patient preference rather than relying solely on indirect comparisons of efficacy endpoints.

### 3.3 The PI3K/AKT/mTOR signaling pathway

The PI3K/AKT/mTOR axis represents one of the common alternative oncogenic pathways in thyroid cancer, particularly in subtypes prone to dedifferentiation or aggressive behavior such as FTC and ATC. This pathway can crosstalk with the MAPK pathway and participate in the development of therapeutic resistance. Aberrant activation of the PI3K/AKT/mTOR pathway therefore provides an important theoretical basis for strategies aimed at combination inhibition and overcoming resistance (9, 97).

#### 3.3.1 The PI3K/AKT/mTOR signaling pathway

The PI3K/AKT/mTOR signaling pathway is a critical intracellular growth-regulatory axis involved in cell proliferation, inhibition of apoptosis, metabolic regulation, and angiogenesis. Within this pathway, activation of PI3K catalyzes the conversion of PIP2 to PIP3, leading to the recruitment and activation of AKT, which subsequently activates the mTOR complex, ultimately regulating protein synthesis and cell-cycle progression (98, 99). mTOR forms two distinct complexes, mTORC1 and mTORC2, which enhance translational and growth signals through downstream effectors such as S6K and 4E-BP1, providing an explanation for the limited efficacy observed with inhibition of individual nodes alone (98, 100). Aberrant activation of this pathway represents an important oncogenic mechanism in multiple malignancies.

In thyroid cancer, activation of this pathway is commonly driven by PTEN loss or mutation, PIK3CA mutations, and AKT overexpression (99, 101). Such abnormalities are frequently observed in FTC, ATC, and a subset of MTC, with particularly high prevalence in ATC, where they are closely associated with tumor progression, loss of differentiation, and therapeutic resistance (99, 102). In addition, activation of this pathway may cooperate with RAS mutations to enhance tumor invasiveness (103). Crosstalk between the PI3K/AKT/mTOR and MAPK pathways allows the PI3K axis to function as a compensatory bypass under therapeutic pressure, thereby contributing to acquired resistance and positioning it as a potential target for combination inhibition strategies (10, 104).

Targeted therapeutic approaches against this pathway have primarily focused on mTOR inhibitors (105). Everolimus and temsirolimus have been approved for other malignancies and have been preliminarily explored in thyroid cancer (106, 107). Preclinical studies and early clinical investigations suggest that everolimus may exerts antiproliferative effects in RAIR-DTC and ATC; several phase I/II studies have reported disease stabilization in a subset of patients, with a PFS of approximately 4–6 months, although objective response rates remain limited and overall efficacy appears more limited than that of approved VEGFR-targeted TKIs (106, 108).

To overcome the limited activity of monotherapy, combination strategies have been increasingly explored, including mTOR inhibitors combined with VEGFR-TKIs (e. g., lenvatinib) or with immune checkpoint inhibitors (e. g., PD-1 antibodies), with preliminary activity signals observed in subtypes such as ATC (102, 106, 109). In addition, PI3K or AKT inhibitors are currently under preclinical evaluation or in early-phase clinical trials, and their role in thyroid cancer remains investigational (110). In clinical practice, assessment of PIK3CA, PTEN, AKT, and RAS co-mutations by NGS may be considered to assist in patient stratification and clinical trial matching, rather than to define an approved PI3K/AKT/mTOR-directed treatment strategy, because PI3K/AKT/mTOR-directed therapy remains exploratory in thyroid cancer and is more appropriately considered as a rational combination partner in aggressive or treatment-resistant disease.

It should be noted that common adverse events associated with mTOR inhibitors include hyperglycemia, hyperlipidemia, and stomatitis, necessitating enhanced monitoring and dose modification when necessary to maintain treatment intensity (111, 112).

### 3.4 Tumor immune regulatory pathways (immune checkpoints)

The overall response to immunotherapy in thyroid cancer exhibits marked heterogeneity; however, in highly aggressive or dedifferentiated subtypes such as ATC and PDTC, immune evasion is more pronounced. In this context, immune checkpoint pathways including PD-1/PD-L1 and CTLA-4 have emerged as important and actionable therapeutic targets. Meanwhile, combination strategies integrating immunotherapy with targeted agents, including VEGFR-TKIs and BRAF/MEK inhibitors, have become a major focus of current research (113, 114).

#### 3.4.1 Immune checkpoints (immune checkpoint inhibition, including PD-1/PD-L1 and CTLA-4)

Programmed cell death protein 1 (PD-1) and its ligand PD-L1 are key immune checkpoint molecules involved in maintaining peripheral immune tolerance (115, 116). Within the tumor microenvironment, overexpression of PD-L1 can bind to PD-1, suppress T-cell activity, and induce T-cell exhaustion, thereby enabling immune evasion (115, 117). In thyroid cancer, high PD-L1 expression is mainly observed in ATC and PDTC, whereas its expression is relatively low in DTC (118). The PD-L1 positivity rate in ATC can exceed 50% and is associated with increased aggressiveness and poor prognosis (119, 120). Compared with DTC, ATC/PDTC often exhibit more prominent immune evasion accompanied by inflammatory responses and immune-cell infiltration; therefore, PD-1/PD-L1 blockade may generate disease control signals in selected subgroups (113, 114).

PD-1/PD-L1 inhibitors such as nivolumab and pembrolizumab, have been approved for the treatment of multiple malignancies (121, 122). In recent years, these agents have also been explored in ATC. Some studies suggest that PD-1 inhibitors may induce tumor shrinkage and disease stabilization in a subset of PD-L1–high ATC patients; although response rates remain heterogeneous, these findings provide a potential therapeutic avenue for this highly lethal tumor type (123, 124).

In addition to PD-1/PD-L1, CTLA-4 (cytotoxic T-lymphocyte–associated antigen 4) is another critical negative immune regulatory pathway that primarily limits immune responses by inhibiting early T-cell activation signals (125, 126). Its inhibitors, such as ipilimumab, have been widely used in malignancies such as melanoma (127). In thyroid cancer, particularly ATC, combination strategies involving PD-1 inhibitors and CTLA-4 inhibitors are under investigation (128). The theoretical rationale is to simultaneously release inhibition at both the T-cell priming/expansion and effector phases, thereby potentially enhancing antitumor immune responses (129, 130).

Clinically, the combination of nivolumab plus ipilimumab has been investigated in a small number of ATC patients. Although sample sizes have been limited, some studies have reported partial responses and even complete responses in advanced ATC, suggesting that a “dual immune checkpoint blockade” strategy may potentially reinvigorate pre-existing but exhausted antitumor immunity within the immunologically “hot” yet checkpoint-suppressed ATC tumor microenvironment (131, 132).

In addition, combination strategies of immune checkpoint inhibitors plus targeted therapy are under active investigation. For example, lenvatinib combined with pembrolizumab has shown preliminary activity signals in RAIR-DTC or ATC (133, 134). Beyond angiogenesis inhibition, VEGFR-TKIs may synergize with PD-1 blockade by remodeling tumor vasculature and the microenvironment and promoting effector T-cell infiltration, thereby potentially increasing response rates and expanding the population that may benefit (135, 136).

Importantly, immune checkpoint inhibition is also being explored in combination with BRAF-directed therapy, particularly in BRAF V600E–mutant ATC. BRAF/MEK inhibition can rapidly suppress MAPK pathway activation and reduce tumor burden, while immune checkpoint blockade may further reactivate antitumor immunity within the immunologically “hot” but checkpoint-suppressed ATC tumor microenvironment, providing a biological rationale for combined targeted and immune therapy (113, 114). In a nonrandomized phase II clinical trial, mutation-directed targeted therapy combined with the anti–PD-L1 antibody atezolizumab was evaluated in ATC. In the BRAF V600E cohort, patients received vemurafenib plus cobimetinib combined with atezolizumab, achieving an objective response rate of 50%, a median progression-free survival of 13.93 months, and a median overall survival of 43.24 months (24). In addition, the FAST multidisciplinary group consensus statement emphasized rapid BRAF V600E testing and timely initiation of BRAF-directed therapy in BRAF V600E–variant ATC. The consensus favored dabrafenib plus trametinib combined with pembrolizumab rather than dabrafenib plus trametinib alone for stage IVB/IVC BRAF V600E–variant ATC when feasible, while also considering the addition of pembrolizumab at progression as an acceptable strategy; however, this recommendation should be interpreted as expert consensus supported by emerging clinical data rather than randomized phase III evidence (25). These findings suggest that immune checkpoint inhibitors combined with BRAF/MEK inhibition may represent a potential treatment strategy for selected patients with BRAF-mutant ATC, although further prospective validation is still needed.

The response to immunotherapy in thyroid cancer is highly heterogeneous, and no widely accepted predictive biomarkers are currently established; the predictive value of PD-L1 expression, tumor mutational burden (TMB), and microsatellite instability (MSI) status is still under evaluation, which limits precise patient selection (137). In clinical practice, close monitoring and management of immune-related adverse events (irAEs), including those that may be amplified in combination regimens, are essential to ensure treatment sustainability (121).

### 3.5 Telomere maintenance and mechanisms of cellular immortalization

Acquisition of telomere maintenance capability is one of the key hallmarks underlying the “unlimited proliferative potential” of tumor cells. In thyroid cancer, TERT promoter mutations do not directly correspond to established targeted therapies; however, they are closely associated with tumor aggressiveness, dedifferentiation, RAI resistance, and increased mortality risk. As such, they are commonly used as important indicators for molecular classification and risk stratification and often cooperate with co-mutations in pathways such as MAPK and PI3K to exert synergistic oncogenic effects (138, 139).

#### 3.5.1 TERT promoter mutations

Telomerase reverse transcriptase (TERT) is a key enzyme responsible for maintaining telomere length at chromosome ends. It plays an essential role in embryonic development and stem cell maintenance, whereas its expression is suppressed in the vast majority of adult somatic cells (140, 141). Mutations in the TERT promoter region—primarily C228T and C250T—can markedly enhance transcriptional activity, leading to sustained TERT expression and conferring “unlimited replicative potential” on tumor cells, thereby promoting malignant transformation and tumor progression (142, 143).

In thyroid cancer, TERT promoter mutations predominantly occur in aggressive subtypes, including ATC, PDTC, FTC, and a subset of high-risk PTC (143). Studies have shown that the mutation rate is approximately 10%–20% in PTC, whereas it can reach 40%–70% in ATC (144, 145). TERT promoter mutations frequently coexist with BRAF V600E or RAS mutations, and their synergistic interaction is thought to markedly accelerate malignant tumor progression (43).

Clinical studies have supported the value of TERT promoter mutations as adverse prognostic biomarkers, which are closely associated with local recurrence, distant metastasis, resistance to radioactive iodine therapy, and disease-related mortality (16, 146). Particularly in BRAF-mutant PTC, the presence of concomitant TERT mutations indicates a more aggressive disease course and poorer prognosis, which may support closer risk-adapted follow-up and individualized management (16, 17).

Although no specific targeted therapies against TERT mutations are currently available, TERT promoter status is mainly used used as an important prognostic biomarker for risk stratification (147). Preclinical studies have explored therapeutic strategies targeting the TERT promoter or downstream regulators of its expression. For example, because TERT expression is regulated by the MAPK pathway, the potential of BRAF/MEK inhibitors to indirectly downregulate TERT expression is under investigation (140, 148).

In addition, agents targeting telomerase activity, such as imetelstat (a telomerase inhibitor), have been studied in other malignancies, but their application in thyroid cancer remains exploratory and unsupported by thyroid cancer-specific clinical evidence (149, 150). Therefore, TERT-directed therapeutic strategies should currently be regarded as investigational rather than established treatment options in thyroid cancer.

### 3.6 Other receptor tyrosine kinase–related signaling pathways and exploratory therapeutic targets

In addition to the MAPK core axis and key pathways such as VEGF and PI3K, thyroid cancer also harbors a number of receptor tyrosine kinase–related abnormalities with lower incidence or still limited evidence, including upregulation of EGFR family signaling and expression or activation of FGFR and IGF-1R in certain subtypes. These targets are more often regarded as “under investigation” or exploratory, and are primarily used to explain mechanisms of therapeutic resistance or to provide candidate directions for subsequent clinical trials.

#### 3.6.1 The EGFR family (rare alterations)

Epidermal growth factor receptor (EGFR) is a member of the ErbB family of receptor tyrosine kinases, which also includes HER2 (ErbB2), HER3 (ErbB3), and HER4 (ErbB4) (151, 152). Under normal physiological conditions, EGFR family receptors regulate cellular proliferation, differentiation, and survival through ligand-dependent activation; in tumors, however, pathway dysregulation can occur through overexpression, mutation, or gene amplification, thereby driving tumor initiation and progression (151, 153). Upon activation, EGFR family receptors can trigger downstream axes such as MAPK and PI3K/AKT/mTOR and engage in crosstalk with resistance-associated pathways. Consequently, in thyroid cancer, EGFR signaling is more often regarded as a candidate bypass pathway related to dedifferentiation and therapeutic resistance (154, 155).

In thyroid cancer, upregulation of EGFR expression is mainly observed in anaplastic thyroid carcinoma (ATC) and in a subset of medullary thyroid carcinoma (MTC), whereas its expression is relatively low in differentiated thyroid cancer (DTC) (156, 157). High EGFR expression has been associated with increased tumor proliferative activity, enhanced angiogenic capacity, and elevated metastatic potential, suggesting a potential role in tumor dedifferentiation (158). It should be emphasized that, in thyroid cancer, EGFR alterations more commonly manifest as expression upregulation rather than frequent classical hotspot mutations; therefore, their clinical significance is primarily reflected in biological phenotypes and as potential entry points for combination therapeutic strategies (159, 160).

Although the mutation frequency of EGFR in thyroid cancer is low, increased EGFR expression has provided a biological rationale for exploratory therapeutic targeting (159, 160). Currently, EGFR-targeted agents mainly include small-molecule TKIs (such as gefitinib, erlotinib, and afatinib) and monoclonal antibodies (such as cetuximab), which have been established in diseases including lung cancer and head and neck squamous cell carcinoma (161, 162).

However, in thyroid cancer, investigations of EGFR inhibitors remain at an early stage. Some preclinical studies suggest that EGFR inhibitors may suppress ATC cell proliferation and induce apoptosis, although the efficacy of monotherapy is limited (156, 163). Several phase I/II clinical studies have explored EGFR inhibitors with chemotherapy, TKIs, or mTOR inhibitors to improve antitumor activity (156, 164). For example, afatinib achieved only transient disease control with low overall response rates in a subset of ATC patients with high EGFR expression (165).

In addition, HER2 has shown a trend toward overexpression in some ATC and MTC tissues, suggesting that HER2 may represent a potential exploratory auxiliary target, although related evidence remains limited (166). In clinical practice, assessment of EGFR and HER2 expression by immunohistochemistry, together with evaluation of gene amplification or accompanying molecular events using FISH or NGS, may help explore identify subgroups more likely to depend on this signaling axis and inform clinical trial enrollment or investigational combination treatment decisions (159, 160).

#### 3.6.2 FGFR/IGF-1R (under investigation)

Fibroblast growth factor receptors (FGFRs) and the insulin-like growth factor 1 receptor (IGF-1R) are receptor tyrosine kinases that play important roles in regulating cell growth, differentiation, migration, and metabolism (167, 168). Both have been shown to possess oncogenic potential in multiple solid tumors and have increasingly been incorporated into exploratory studies of targeted therapies in thyroid cancer. Unlike well-defined fusion-driven alterations such as RET or NTRK, abnormalities involving FGFR and IGF-1R in thyroid cancer are more commonly manifested as expression upregulation or pathway activation. These alterations are often invoked to explain dedifferentiation, invasiveness, and resistance bypass mechanisms, and to provide candidate directions for investigational combination therapies or clinical trial development (169, 170).

The FGFR family comprises four subtypes (FGFR1–4). Their activation can occur through ligand binding as well as gene amplification, mutation, or fusion, subsequently activating downstream signaling pathways such as MAPK, PI3K/AKT, and JAK/STAT (168, 171). In thyroid cancer, particularly in anaplastic thyroid carcinoma (ATC) and RAI-refractory DTC, upregulation of FGFR1 and FGFR4 expression has been observed and has been associated with enhanced proliferative and metastatic capacity (169, 172, 173). Although high-frequency FGFR fusions or mutations are uncommon, FGFR overexpression provides a theoretical basis for exploratory therapeutic intervention (169, 174). Small-molecule inhibitors such as erdafitinib and AZD4547 have been used in other malignancies, while their role in thyroid cancer remains under early-stage evaluation (174, 175).

Upon binding to its ligand IGF-1, IGF-1R activates the PI3K/AKT/mTOR and MAPK pathways, promoting cell proliferation and resistance to apoptosis. IGF-1R expression has been reported to be upregulated in differentiated thyroid cancer and MTC and may be associated with treatment resistance, particularly failure of radioactive iodine therapy, possibly through downregulation of NIS expression leading to impaired iodine uptake (176, 177).

With regard to targeted agents, IGF-1R inhibitors such as linsitinib and BMS-754807 have entered phase I/II clinical trials in several malignancies, but their thyroid cancer-specific evidence remains sparse (178, 179). However, clinical evidence supporting their use in RAIR-DTC or ATC remains limited, and no corresponding indications have been approved to date.

Given that FGFR and IGF-1R alterations in thyroid cancer more often reflect pathway activation rather than single driver mutations, practical approaches may include immunohistochemical assessment of FGFR1/FGFR4 and IGF-1R expression, combined with NGS and CNV analyses to evaluate amplification and co-mutation backgrounds. Such strategies may aid clinical trial enrollment or exploration of investigational combination approaches, for example with VEGFR-TKIs or PI3K/mTOR pathway inhibitors (169, 170).

## 4 Emerging targets and investigational agents

With the rapid advances in molecular biology and gene sequencing technologies, precision classification of thyroid cancer has continued to deepen, and a series of emerging targets and therapeutic strategies are entering early-phase clinical validation, demonstrating notable potential particularly in RAI-refractory thyroid cancer, ATC, and recurrent or metastatic MTC.

### 4.1 Combination targeted therapy strategies

An important trend in targeted therapy is dual or multi-pathway inhibition. In BRAF V600E–mutant ATC, combination therapy with a BRAF inhibitor (such as dabrafenib) and a MEK inhibitor (such as trametinib) has demonstrated clinically meaningful antitumor activity and has been approved by the FDA for this indication. This combination strategy simultaneously suppresses upstream and downstream components of the MAPK pathway, thereby helping to delay or reduce MAPK pathway reactivation associated with single-agent treatment (22, 180).

In addition, the combination of BRAF/MEK inhibitors with PD-1 immune checkpoint inhibitors has shown biological and early clinical rationale for enhancing antitumor immunity and is being evaluated in clinical studies. Such “targeted therapy plus immunotherapy” combinations remain an active investigational direction for ATC and selected RAIR-DTC settings (181, 182).

In MTC, although RET inhibitors such as selpercatinib and pralsetinib have shown favorable efficacy, acquired resistance mutations, particularly solvent-front mutations such as G810S/G810R, have been reported (183, 184). To address this issue, next-generation RET inhibitors are being developed to target resistance-associated RET alterations (185). Among them, TPX-0046 is a structurally differentiated macrocyclic RET/SRC inhibitor designed to retain activity against diverse RET alterations, including solvent-front mutations. Preclinical studies showed that TPX-0046 inhibited RET phosphorylation and RET-driven cell proliferation, demonstrated activity against the G810R solvent-front mutation, and produced antitumor activity in RET-driven xenograft models (186). However, subsequent in-silico analysis suggested that TPX-0046 may remain vulnerable to bulky gatekeeper V804 mutations, L881F, or S891L-related resistance, indicating that resistance monitoring and further inhibitor optimization remain necessary (187). In addition to TPX-0046, other next-generation RET inhibitors, such as SY-5007 and APS03118, have also shown preliminary activity against RET-altered tumors or resistance-associated RET mutations in early clinical or preclinical studies (188, 189). These findings suggest that next-generation RET inhibitors may provide potential therapeutic opportunities after resistance to current selective RET inhibitors, although their optimal sequencing, durability of response, long-term efficacy, and safety still require further clinical validation.

### 4.2 Exploration of novel therapeutic targets: CDKs, AURKA, and others

In recent years, with the continued deepening of research into the molecular mechanisms of thyroid cancer, a number of emerging oncogenic pathways beyond traditional targets have gradually been elucidated.

Among these, Aurora kinase A (AURKA) has attracted increasing attention because of its central role in cell-cycle regulation and chromosome segregation (190, 191). Studies have reported that AURKA expression is significantly upregulated in PTC and is associated with lymph node metastasis, TNM stage, and unfavorable prognosis (192, 193). Zhao et al. (2022) reported that AURKA directly binds to SIN1 and inhibits its ubiquitin-dependent degradation, leading to aberrant activation of the mTORC2–AKT signaling pathway and thereby enhancing tumor cell proliferation and migratory capacity (192). Another study suggested that AURKA inhibition reduces PFKFB3-dependent glycolytic metabolism and increases the sensitivity of thyroid cancer cells to multikinase TKIs (such as sorafenib), suggesting that AURKA inhibitors may enhance existing therapeutic efficacy through metabolic reprogramming and signaling synergy (193).

Although no AURKA inhibitors have yet been approved for thyroid cancer, its high expression and functional activity in several aggressive subtypes, including ATC, PDTC, and some progressive PTC, support AURKA as a potential investigational target rather than an established therapeutic option (191). AURKA inhibitors that have already entered clinical studies in other solid tumors, such as alisertib (MLN8237), could be considered candidate agents for future validation in thyroid cancer.

CDK4/6 inhibitors target the core cell cycle regulatory axis (Cyclin D–CDK4/6–RB), which promotes the G1/S transition through phosphorylation of the RB protein and has been therapeutically validated in multiple solid tumors (194, 195). In thyroid cancer, however, the evidence supporting CDK4/6 inhibitors should be interpreted more cautiously. Although dysregulation of cell cycle–related pathways and upstream oncogenic signaling, such as MAPK and PI3K/AKT pathways, provides a biological rationale for CDK4/6 inhibition, thyroid cancer-specific therapeutic evidence remains limited and is mainly derived from preclinical studies in ATC, PDTC, or other advanced/dedifferentiated thyroid cancer models rather than from prospective clinical trials across major histological subtypes (196–198). In a translational study of advanced thyroid carcinomas, CDK4 phosphorylation was detected in all well-differentiated thyroid carcinomas analyzed, 19/20 PDTCs, 16/23 ATCs, and 18/21 thyroid cancer cell lines, whereas absence of phosphorylated CDK4 was associated with CDK4/6 inhibitor insensitivity; importantly, palbociclib showed synergistic growth suppression when combined with BRAF/MEK inhibition in thyroid cancer cell lines (196). In ATC models, palbociclib inhibited proliferation in RB1-wild-type ATC cells and xenografts, but resistance emerged rapidly; combined PI3K/mTOR and CDK4/6 inhibition prolonged and potentiated palbociclib activity, supporting CDK4/6 blockade mainly as a potential combination partner in aggressive ATC (197). Limited preclinical evidence has also suggested potential relevance in selected BRAF V600E-mutant, CDKN2A/P16-deficient RAIR PTC models, where vemurafenib plus palbociclib was reported to overcome vemurafenib resistance (199). By contrast, evidence in FTC remains insufficient for subtype-specific therapeutic conclusions, and MTC data are not favorable for CDK4/6-specific inhibition, because palbociclib reduced proliferation only modestly and was not cytotoxic in MTC cells, whereas CDK7/CDK9-centered transcriptional targeting appeared more active (196, 200). From a translational perspective, CDK4/6 inhibition in thyroid cancer may therefore be more relevant to biomarker-guided combination strategies than to broad single-agent application, especially with MAPK or PI3K/mTOR pathway inhibition in ATC/PDTC and other aggressive dedifferentiated tumors. In addition, although CDK4/6 inhibitors have demonstrated potential synergy with immunotherapy in other malignancies, possibly by modulating tumor antigen presentation, cytokine milieu, or antitumor immune responses (201–203), such combinations remain hypothesis-generating in thyroid cancer and require thyroid cancer-specific validation. CDK4/6 inhibitors have not yet received approved indications in thyroid cancer; current evidence supports cautious discussion of this target as an exploratory strategy, particularly for ATC and selected aggressive molecular contexts, rather than as a broadly validated therapeutic approach for all thyroid cancer subtypes (196, 197).

### 4.3 Redifferentiation strategies and restoration of radioiodine sensitivity

In a subset of RAI-refractory PTC patients, loss of NIS expression or abnormal subcellular localization can result in failure of RAI therapy (204, 205). In recent years, investigators have attempted “redifferentiation therapy” strategies to reactivate NIS expression pharmacologically, enabling tumors to regain iodine uptake capacity and restore sensitivity to RAI (204, 205).

MEK inhibitors (selumetinib) and histone deacetylase (HDAC) inhibitors (such as panobinostat) have been shown to upregulate NIS expression and induce re-uptake of RAI, and a small number of patients have successfully undergone RAI therapy after redifferentiation treatment. This strategy is particularly applicable to RAS-mutant RAIR-DTC without BRAF mutations and has entered phase I/II clinical validation (204, 206).

### 4.4 Precision molecular profiling and individualized treatment

The patterns of mutual exclusivity and co-mutations among different driver alterations in thyroid cancer (such as BRAF, RAS, RET, TERT, and PI3K) are complex, and they influence responses to TKIs, RAI, and immunotherapy (17, 207). Therefore, molecular classification systems based on multigene testing using NGS platforms are being progressively implemented. For patients without actionable target alterations, precision treatment possibilities may be introduced through basket-trial approaches (such as NCI-MATCH and TARGET) (208); for patients with a risk of recurrence despite low-grade initial pathology, early-intervention strategies based on co-mutations such as TERT/BRAF are being explored (17).

### 4.5 Clinical indications for genetic/molecular analysis in thyroid cancer

In clinical practice, genetic or molecular analysis of thyroid cancer tissue should be performed when the results are expected to influence diagnosis, risk stratification, treatment selection, or clinical trial enrollment. Molecular testing is not required for every patient with low-risk, localized differentiated thyroid cancer after complete surgical resection; rather, it should be prioritized in patients with advanced, recurrent, metastatic, progressive, radioactive iodine–refractory, or histologically aggressive disease, as well as in patients for whom systemic targeted therapy is being considered (209–213).

For differentiated thyroid cancer, particularly RAIR-DTC, molecular profiling is recommended to identify actionable alterations such as BRAF V600E, RET fusions, NTRK fusions, ALK fusions, RAS mutations, TERT promoter mutations, and PI3K-axis alterations. These results may guide genotype-matched therapy, support selection between selective inhibitors and VEGFR-centered multikinase TKIs, assist in clinical trial matching, and provide prognostic information when adverse co-mutation patterns such as BRAF/TERT or RAS/TERT are detected (16, 17, 209–211, 214). In patients with progressive disease after initially iodine-avid tumors, molecular testing should also be integrated with functional assessment of iodine avidity when redifferentiation therapy is being considered (26, 215–217).

In aggressive histological subtypes, especially ATC and PDTC, molecular testing should be performed as early as possible because the therapeutic window is often narrow and treatment decisions must be made rapidly. At minimum, testing should include BRAF V600E and clinically actionable fusions such as RET and NTRK, and broader NGS panels are preferable when tissue and turnaround time permit (211, 212). Early identification of BRAF V600E can support prompt initiation of BRAF/MEK inhibition in ATC, whereas detection of RET or NTRK fusions may allow the use of corresponding selective inhibitors (22, 47, 55, 58, 212). Testing for additional alterations, including TP53, TERT promoter mutations, PI3K/AKT/mTOR pathway alterations, and other resistance-related events, may further assist prognostic assessment and clinical trial selection (207, 212, 218, 219).

For medullary thyroid carcinoma, RET testing has particular clinical importance. Because MTC is frequently driven by RET alterations and does not respond to radioiodine therapy, molecular testing should be performed in patients with advanced, recurrent, metastatic, or progressive disease to guide the use of selective RET inhibitors or multikinase TKIs (40, 211, 213, 220). In addition, germline RET testing should be distinguished from somatic tumor testing when hereditary MTC or MEN2 is suspected, because germline findings have implications for genetic counseling, family screening, and long-term surveillance (40, 211).

From a technical perspective, the testing strategy should be selected according to the clinical question and sample availability. DNA-based NGS panels are useful for detecting point mutations, small insertions/deletions, copy-number alterations, and some fusions; however, because clinically relevant fusions involving RET, NTRK, ALK, and other kinases may be missed by limited DNA-based assays, RNA-based fusion testing or combined DNA/RNA panels should be considered when fusion-positive disease is suspected or when no driver alteration is identified by DNA testing alone (68, 70, 211, 214, 221). Molecular testing can be performed on surgical specimens, biopsy tissue, or selected fine-needle aspiration samples when adequate tumor material is available (221). At disease progression after targeted therapy, repeat molecular profiling should be considered to identify acquired resistance mechanisms, such as RET solvent-front mutations or bypass pathway activation, and to guide subsequent treatment sequencing or trial enrollment (183–185, 211).

Overall, molecular analysis should be viewed as a clinically actionable tool rather than a purely descriptive investigation. Its greatest value lies in patients with advanced or treatment-refractory disease, aggressive histology, suspected actionable alterations, or planned targeted therapy. Integrating histological subtype, disease status, molecular results, functional imaging, treatment availability, and multidisciplinary discussion can help translate genomic information into practical and individualized therapeutic decisions.

## 5 Summary of subtype-specific targeted therapeutic strategies

Distinct pathological subtypes of thyroid cancer differ in molecular driver events, signaling pathway dependencies, and clinical behavior. However, because the biological mechanisms and representative agents of each pathway have been discussed in detail above, this section provides only a concise subtype-oriented synthesis of therapeutic decision-making, with the main practical strategies summarized in **Table 2**. In general, targeted therapy should follow the principle of “histological subtype as the clinical framework and molecular classification as the therapeutic core,” especially in patients with radioactive iodine-refractory disease, disease progression, or advanced-stage tumors (9, 10).

**Table 2:** Targeted therapeutic strategies across major pathological subtypes of thyroid cancer.

Pathological subtype	Key molecular drivers and pathway characteristics	Therapeutic strategy overview (preferred, alternative, and investigational)
DTC: PTC and FTC (including RAIR-DTC)	PTC is more frequently associated with MAPK pathway alterations, including BRAF V600E, RET fusions, NTRK fusions, and a subset of RAS mutations. FTC more commonly involves RAS mutations and alterations in the PI3K/AKT/mTOR axis. In the RAIR-DTC setting, systemic treatment is generally guided more by actionable molecular alterations and disease behavior than by histological subtype alone.	Established/guideline-supported options: For RET fusion–positive tumors, selective RET inhibitors such as selpercatinib or pralsetinib may be considered; for NTRK fusion–positive tumors, larotrectinib or entrectinib may be used as genotype-matched therapy. For progressive RAIR-DTC without a highly actionable driver alteration, or when genotype-matched therapy is not selected, VEGFR-centered multikinase inhibitors, such as lenvatinib or sorafenib, remain standard systemic options. Conditional/individualized options: For BRAF V600E–mutant RAIR-PTC, BRAF-targeted therapy with or without MEK inhibition may be considered after individualized assessment; however, current evidence does not demonstrate superiority over VEGFR-centered multikinase inhibitors in the first-line setting, and this strategy should not be designated as preferred over MKIs. Investigational strategies: Redifferentiation strategies, including MAPK pathway inhibition–based approaches; combinations of VEGFR-TKIs with immune checkpoint inhibitors; and strategies targeting bypass pathways such as FGFR, IGF-1R, or PI3K/mTOR.
ATC (including PDTC and dedifferentiated spectrum)	Characterized by aggressive clinical behavior, genomic instability, and frequent alterations such as BRAF V600E, TERT promoter mutations, TP53 mutations, PI3K-axis alterations, and occasionally actionable fusions such as RET or NTRK. Immune checkpoint activation and angiogenic signaling may also contribute to therapeutic vulnerability.	Established/guideline-supported options: For BRAF V600E–mutant tumors, dabrafenib plus trametinib remains the most established targeted option. For tumors harboring actionable RET or NTRK fusions, corresponding selective inhibitors should be prioritized when clinically feasible. Conditional/individualized options: VEGFR-centered multikinase TKIs, such as lenvatinib, may be considered in selected patients; immune checkpoint inhibitors may be considered in selected contexts, particularly as part of combination strategies or clinical trials. Investigational strategies: Combination strategies including BRAF/MEK inhibition plus immune checkpoint blockade, VEGFR-TKIs plus PD-1/PD-L1 blockade, and agents targeting PI3K/mTOR, AURKA, CDK4/6, or other resistance-related pathways.
MTC	Predominantly driven by activating RET mutations, particularly in hereditary disease and a substantial proportion of sporadic cases. MTC arises from parafollicular C cells and is independent of radioiodine uptake.	Established/guideline-supported options: For RET-mutant MTC, selective RET inhibitors such as selpercatinib or pralsetinib are preferred options when available and appropriate. Conditional/individualized options: Multikinase TKIs such as vandetanib or cabozantinib may be considered when selective RET inhibitors are unavailable, unsuitable, or after resistance develops. Investigational strategies: Next-generation RET inhibitors designed to overcome acquired resistance, including agents targeting RET solvent-front or other resistance-associated mutations, such as TPX-0046 and related next-generation inhibitors. Current evidence supporting immunotherapy in MTC remains limited.

**Table 2** summarizes clinically relevant targets, corresponding targeted agents, and their classification as Preferred, Alternative, or Investigational across the major pathological subtypes, providing a practical reference for clinical decision-making and comparison. **Table 3** further complements this subtype-oriented overview by comparing representative targeted therapeutic strategies in terms of molecular indication or clinical setting, key efficacy outcomes, common toxicities, and evidence status.

**Table 3:** Clinical comparison of representative targeted therapeutic strategies in thyroid cancer.

Therapeutic strategy	Molecular indication/clinical setting	Representative agents	Key efficacy outcomes	Common toxicities	Evidence status
VEGFR-centered multikinase inhibition	Progressive RAIR-DTC without highly actionable driver alterations or when genotype-matched therapy is not selected	Lenvatinib, sorafenib	Lenvatinib improved median PFS to 18.3 months versus 3.6 months with placebo in SELECT; sorafenib improved median PFS to 10.8 months versus 5.8 months with placebo in DECISION	Hypertension, proteinuria, fatigue, diarrhea, hand–foot skin reaction	Established standard options for RAIR-DTC; no direct head-to-head trial proves superiority between agents
Subsequent-line VEGFR/MET/AXL inhibition	RAIR-DTC after progression on prior VEGFR-targeted therapy	Cabozantinib	COSMIC-311 showed improved median PFS of 11.0 months versus 1.9 months with placeb	Hypertension, diarrhea, fatigue, palmar–plantar erythrodysesthesia, mucosal inflammation	Established subsequent-line option after prior lenvatinib and/or sorafenib
BRAF/MEK inhibition	BRAF V600E–mutant ATC	Dabrafenib plus trametinib	The ROAR basket study showed clinically meaningful antitumor activity in BRAF V600E–mutant ATC	Pyrexia, rash, fatigue, diarrhea, cardiomyopathy, ocular toxicity	FDA-approved option for BRAF V600E–mutant ATC; evidence mainly from basket/single-arm data
BRAF/MEK inhibition plus immunotherapy	Selected BRAF V600E–mutant ATC	Vemurafenib plus cobimetinib plus atezolizumab; dabrafenib plus trametinib plus pembrolizumab-based strategies	A nonrandomized phase II trial reported ORR of 50%, median PFS of 13.93 months, and median OS of 43.24 months in the BRAF V600E cohort	Targeted therapy-related rash, fever, diarrhea, liver enzyme elevation, plus immune-related adverse events	Promising but still investigational; requires further prospective validation
Selective RET inhibition	RET-mutant MTC or RET fusion–positive thyroid cancer	Selpercatinib, pralsetinib	LIBRETTO-531 showed improved PFS with selpercatinib compared with cabozantinib or vandetanib in advanced RET-mutant MTC; median PFS was not reached versus 16.8 months in the control group	Hypertension, dry mouth, diarrhea, edema, elevated transaminases, QT prolongation	Established genotype-matched therapy, especially strong evidence in RET-mutant MTC
TRK inhibition	NTRK fusion–positive thyroid carcinoma	Larotrectinib, entrectinib	Larotrectinib showed ORR exceeding 70% in NTRK fusion–positive thyroid carcinoma; entrectinib showed ORR of approximately 57% across NTRK fusion–positive solid tumors	Fatigue, dizziness, nausea, weight gain, dysgeusia, elevated transaminases	Tumor-agnostic approved options; thyroid-specific cohorts remain relatively small
PI3K/AKT/mTOR pathway inhibition	Selected aggressive or treatment-resistant thyroid cancers with PI3K-axis alterations	Everolimus, temsirolimus	Early-phase studies reported disease stabilization in selected patients, but objective responses were limited and PFS was generally shorter than with approved VEGFR-TKIs	Stomatitis, hyperglycemia, hyperlipidemia, cytopenia, pneumonitis	Investigational in thyroid cancer; more suitable as a combination partner than established monotherapy
Next-generation inhibitors for acquired resistance	RET or NTRK inhibitor resistance mutations, including solvent-front alterations	TPX-0046, SY-5007, APS03118, vepafestinib, repotrectinib	Preclinical or early clinical data suggest potential activity against resistance-associated RET or TRK alterations	Agent-specific toxicity profiles remain under evaluation	Investigational or emerging strategy; optimal sequencing and durability require further validation

### 5.1 Differentiated thyroid carcinoma: PTC and FTC

Differentiated thyroid carcinoma (DTC), mainly including papillary thyroid carcinoma (PTC) and follicular thyroid carcinoma (FTC), shares substantial overlap in systemic treatment principles, particularly in the setting of radioactive iodine-refractory disease (RAIR-DTC). Therefore, targeted therapy for PTC and FTC is more appropriately guided by actionable molecular alterations and disease behavior rather than by histological subtype alone. Nevertheless, PTC and FTC exhibit different dominant molecular patterns: PTC is more frequently associated with MAPK pathway activation, including BRAF V600E, RET fusions, and NTRK fusions, whereas FTC more commonly involves RAS mutations and PI3K/AKT/mTOR-axis alterations (9, 209, 222, 223).

For patients with RET fusion–positive DTC, selective RET inhibitors such as selpercatinib or pralsetinib should be considered; for NTRK fusion–positive tumors, larotrectinib or entrectinib may be used as genotype-matched treatment options (47, 55, 58, 214, 224). For patients without clearly targetable drivers, without highly actionable fusions, or in whom genotype-matched therapy is not selected, VEGFR-centered multikinase TKIs remain the cornerstone of systemic therapy for RAIR-DTC, with lenvatinib and sorafenib as the mainstay options (209, 225).

In RAIR-DTC or progressive PTC with BRAF V600E, BRAF-targeted therapy with or without MEK inhibition may be considered as a genotype-matched option after individualized assessment. However, current ASCO guidance does not establish BRAF-targeted therapy as superior to VEGFR-centered multikinase inhibitors in the first-line setting; rather, it supports its use as a conditional option based on low-quality evidence (210). Therefore, BRAF-targeted therapy should be presented as an individualized option rather than as a preferred first-line strategy over multikinase inhibitors in BRAF V600E-mutant RAIR-PTC.

Redifferentiation therapy may be considered in selected patients with RAIR-DTC, particularly when MAPK pathway inhibition is expected to restore iodine avidity. Combination with a MEK inhibitor may enhance pathway suppression and may contribute to redifferentiation by restoring NIS expression and/or membrane localization in selected tumors (26, 226). In clinical redifferentiation studies, restoration of iodine avidity has generally been evaluated using functional radioiodine uptake assays, such as quantitative ^124^I PET/CT lesional dosimetry or rhTSH-stimulated diagnostic radioiodine whole-body scanning after MAPK-pathway inhibition (215, 216). Because NIS functionality depends not only on SLC5A5/NIS expression but also on protein expression, membrane localization, and functional iodine uptake, it should not be inferred from DNA-based NGS findings alone (217).

In clinical practice, molecular profiling using NGS panels that cover relevant mutations and gene fusions is recommended to identify actionable genomic drivers and guide genotype-matched therapy, with RNA-based fusion testing added when DNA-based panels are insufficient (211, 214, 221). However, DNA-based NGS should be used primarily to identify actionable alterations and should not be presented as a routine assay for detecting NIS downregulation. When assessment of NIS-related dedifferentiation is needed, complementary assays may include SLC5A5/NIS mRNA expression analysis, such as qRT-PCR or transcriptomic approaches, NIS protein expression and membrane localization by immunohistochemistry, and functional assessment of iodine avidity using diagnostic ^123^I/^131^I radioiodine scanning or quantitative ^124^I PET/CT (215–217).

Because many clinical studies have enrolled broader RAIR-DTC populations rather than FTC-specific cohorts, therapeutic strategies for FTC should be interpreted as molecularly guided approaches applicable to selected DTC patients rather than as evidence uniquely specific to FTC. For patients with initially good iodine uptake who subsequently experience disease progression, dynamic reassessment of RAI sensitivity is warranted to determine the appropriateness of redifferentiation approaches or transition to systemic therapy (36, 222).

### 5.2 ATC

ATC is characterized by an aggressive clinical course and a narrow therapeutic window. Because its molecular mechanisms, immune microenvironment, and exploratory targets have been discussed in previous sections, subtype-based management should focus on rapid molecular testing and timely treatment selection rather than repeated mechanistic explanation. In clinical practice, early testing for BRAF V600E and actionable fusion events, including RET and NTRK fusions, is essential to guide first-line precision therapy (212, 218, 219).

For BRAF V600E–positive ATC, dabrafenib plus trametinib remains the most established targeted option and has received FDA approval (22, 212). If actionable RET or NTRK fusions are identified, corresponding selective inhibitors should be prioritized (47, 55, 58, 212). Immunotherapy may also be considered in selected patients, particularly in the context of PD-L1 expression, inflammatory infiltration, or combination strategies. Current investigational directions include immune checkpoint inhibitors combined with VEGFR-TKIs or BRAF/MEK inhibitors, as well as exploratory approaches targeting PI3K/mTOR, AURKA, and CDK4/6 (24, 227, 228).

Therefore, ATC management should emphasize “seizing the therapeutic window.” Rapid molecular testing should be completed as early as possible, and treatment decisions should integrate actionable alterations, disease burden, performance status, feasibility of combination therapy, and clinical trial availability (25, 211).

### 5.3 MTC

MTC originates from parafollicular C cells and does not concentrate iodine, rendering RAI ineffective; therefore, systemic treatment is primarily organized around RET-driven inhibition (229). Upfront RET testing is recommended, with differentiation between germline and somatic alterations and assessment of MEN2 risk when necessary (211).

For RET-mutant MTC, selective RET inhibitors such as selpercatinib and pralsetinib are preferred options because of their efficacy and tolerability. When these agents are unavailable, unsuitable, or after resistance develops, multikinase TKIs such as vandetanib or cabozantinib may be considered (45, 213, 220). With the increasing use of selective RET inhibitors, resistance mutations such as RET G810-series alterations have emerged, and next-generation RET inhibitors, including TPX-0046, are being developed to address acquired resistance (184–187, 230). Upon disease progression, molecular re-profiling is advised to identify resistance-associated alterations and to support enrollment in clinical trials or selection of subsequent targeted strategies (211).

Overall, DTC treatment is mainly guided by actionable alterations, VEGFR-centered systemic therapy, and redifferentiation potential; ATC requires rapid molecular testing and early initiation of matched or combination therapy; and MTC is centered on RET-directed treatment. By referring to **Table 2**, clinicians can integrate histological subtype, molecular profiling, treatment availability, and patient condition into a more individualized targeted therapeutic strategy.

## 6 Future perspectives

Although targeted therapy has substantially improved the management of advanced thyroid cancer, future progress will depend on the deeper integration of molecular diagnostics, next-generation drug development, rational combination strategies, and more equitable implementation of precision medicine. First, next-generation sequencing (NGS) should be increasingly incorporated into routine care for patients with advanced, progressive, RAI-refractory, or high-risk thyroid cancer. Comprehensive molecular profiling can identify actionable alterations such as BRAF, RET, NTRK, ALK, RAS, TERT promoter mutations, and PI3K-axis alterations, thereby guiding genotype-matched therapy, supporting clinical trial enrollment, and informing treatment sequencing (68, 70, 209, 211, 214, 221). In particular, fusion detection should be emphasized, because RET, NTRK, and ALK fusions may be missed by limited DNA-based assays; therefore, RNA-based fusion testing or DNA/RNA combined panels should be considered when clinically feasible (68, 70, 211, 214, 221). In addition, dynamic molecular reassessment at disease progression may help identify acquired resistance mechanisms and guide subsequent targeted therapy or trial participation (184, 185, 211). Because the strength of evidence varies substantially across targets and pathological subtypes, established standards of care, guideline-supported genotype-matched options, and investigational strategies should be clearly distinguished in clinical decision-making.

Second, the development of next-generation inhibitors targeting resistance mutations represents a major future direction. Acquired resistance remains a key limitation of targeted therapy, arising from secondary target mutations, bypass pathway activation, lineage plasticity, and tumor heterogeneity (102, 183–185). In RET-altered thyroid cancer, resistance-associated alterations such as RET solvent-front G810-series mutations have been reported after selective RET inhibition, highlighting the need for next-generation RET inhibitors (183–185). Agents such as TPX-0046 and other emerging RET inhibitors, including SY-5007, APS03118, and vepafestinib, have shown potential activity against RET-altered tumors or resistance-associated RET mutations in preclinical or early clinical settings, although their optimal sequencing, long-term efficacy, and safety still require prospective validation (186–189, 230). Similarly, for NTRK fusion–positive tumors, next-generation TRK inhibitors such as repotrectinib may provide therapeutic opportunities after resistance to first-generation TRK inhibitors, particularly in the setting of solvent-front mutations (60–62). These developments indicate that future targeted therapy should shift from static first-line matching toward adaptive treatment strategies guided by resistance evolution.

Third, rational combination therapy will be essential to overcome compensatory signaling and immune escape. Rather than empirically combining multiple agents, future regimens should be designed according to tumor biology, pathway dependency, and toxicity tolerance. For BRAF V600E–mutant ATC, BRAF/MEK inhibition combined with immune checkpoint blockade is a particularly important direction, because MAPK inhibition may rapidly reduce tumor burden, whereas immunotherapy may further reactivate antitumor immunity in the checkpoint-suppressed tumor microenvironment (24, 25, 113, 114). In RAIR-DTC and ATC, VEGFR-TKIs combined with PD-1/PD-L1 blockade may exert synergistic effects by remodeling tumor vasculature, reducing immunosuppressive features of the microenvironment, and promoting effector T-cell infiltration (133–136). Other potential strategies, such as MAPK pathway inhibition combined with VEGFR-centered TKIs, PI3K/mTOR inhibition, or cell-cycle–targeted agents, may help suppress bypass activation and delay resistance, but these approaches require careful evaluation because of the risk of additive toxicity (102, 109, 196, 197). Therefore, optimization of treatment sequencing, dose adjustment, patient selection, and adverse-event prediction will be critical for improving the therapeutic index of combination regimens.

Fourth, predictive biomarkers and tumor microenvironment profiling should be further developed to guide individualized therapy. At present, the predictive value of single biomarkers remains limited. For example, PD-L1 expression, tumor mutational burden, and microsatellite instability may provide some information for immunotherapy selection, but their clinical utility in thyroid cancer remains heterogeneous and incompletely established (113, 137). Future biomarker systems should integrate multiple dimensions, including driver alterations, co-mutation patterns such as BRAF/TERT or RAS/TERT, NIS/SLC5A5 expression, NIS protein localization, functional iodine uptake, angiogenesis-related signatures, and immune microenvironment features (16, 17, 114, 137, 176, 204, 205, 209). Tumor microenvironment profiling, including assessment of immune-cell infiltration, PD-L1 expression, inflammatory signaling, vascular normalization, and angiogenesis-related pathways, may help identify patients more likely to benefit from immune checkpoint blockade or VEGFR-TKI–based combinations (113, 114, 133–136). Such multidimensional biomarkers may also support toxicity prediction and assist in selecting patients for rational combination therapy.

Fifth, redifferentiation therapy remains an important future strategy for selected patients with RAIR-DTC. Loss of iodine avidity is closely related to impaired NIS expression, abnormal membrane localization, and MAPK pathway activation (176, 204, 205). Pharmacological inhibition of MAPK signaling, particularly with MEK inhibitors or BRAF/MEK-directed strategies in appropriate molecular contexts, may restore iodine uptake and enable renewed RAI therapy in a subset of patients (26, 204, 205). Clinical studies have used functional assays such as diagnostic radioiodine scanning and quantitative ^124^I PET/CT lesional dosimetry to evaluate restored iodine avidity after redifferentiation therapy (215, 216). Future research should further clarify which molecular subgroups are most likely to benefit, how long targeted therapy should be administered before RAI, how restored uptake should be quantified, and how genomic profiling, NIS-related assays, and functional imaging can be integrated into practical redifferentiation algorithms (26, 204, 205, 215, 216).

Finally, the global applicability of precision therapy must also be considered. Although NGS-based molecular classification and genotype-matched therapy are increasingly available in specialized centers, their broader implementation remains constrained by cost and reimbursement issues, sequencing and molecular pathology infrastructure, turnaround time, access to RNA-based fusion testing, availability of targeted agents, and the need for multidisciplinary molecular tumor boards (208, 209, 231). These barriers are particularly relevant in low-resource settings, where limited laboratory capacity, insufficient bioinformatics support, unequal access to molecular pathology expertise, ethical and data-sharing concerns, and financial constraints may prevent patients from receiving timely genomic testing or matched therapy (231, 232). To improve global applicability, future efforts should focus on developing cost-effective and standardized testing workflows, prioritizing high-yield molecular assays for patients with advanced or high-risk disease, establishing regional referral networks, expanding virtual molecular tumor boards, strengthening local bioinformatics and molecular pathology capacity, improving reimbursement policies, and generating real-world evidence across diverse healthcare systems (208, 211, 231, 232). These measures may help ensure that advances in thyroid cancer precision medicine are translated not only in high-resource academic centers but also in broader clinical settings worldwide.

## 7 Conclusion

Targeted therapy has substantially reshaped the therapeutic landscape of thyroid cancer, particularly for patients with advanced, recurrent, radioiodine-refractory, or highly aggressive disease. With the increasing understanding of molecular alterations in thyroid cancer, treatment strategies have gradually shifted from empiric multikinase inhibition toward molecularly matched and subtype-adapted precision therapy. Actionable alterations such as BRAF V600E, RET mutations or fusions, and NTRK fusions have provided clinically meaningful therapeutic opportunities, while VEGFR-centered multikinase inhibitors remain important systemic options for patients with RAIR-DTC who lack highly actionable driver alterations or are unsuitable for genotype-matched therapy.

Across pathological subtypes, targeted therapeutic decision-making should be guided by both histological context and molecular profiling. In DTC, systemic therapy is mainly organized around MAPK pathway activation, angiogenesis inhibition, and restoration of radioiodine sensitivity. In ATC, rapid molecular testing and timely initiation of matched therapy are particularly important because of the narrow therapeutic window and aggressive clinical course. In MTC, RET-driven treatment has become central to systemic management, especially with the clinical application of selective RET inhibitors.

Overall, targeted therapy in thyroid cancer is moving toward a more individualized, dynamically reassessed, and multidisciplinary treatment model. Continued integration of molecular testing, rational treatment sequencing, adverse-event management, and clinical trial participation will be essential to further improve long-term disease control and patient outcomes.
